# Data on spectrum-based fluorescence resonance energy transfer measurement of *E. coli* multidrug transporter AcrB

**DOI:** 10.1016/j.dib.2018.10.155

**Published:** 2018-11-03

**Authors:** Yuguang Cai, Thomas Wilkop, Yinan Wei

**Affiliations:** a Department of Chemistry, University of Kentucky, Lexington, KY 40506, United States; b Light Microscopy Core, University of Kentucky, Lexington, KY 40536, United States

## Abstract

This paper presented the dataset of correction parameters used in the determination of the energy transfer efficiencies from the spectrum-based fluorescence resonance energy transfer (FRET) measurement in a trimeric membrane protein AcrB. The cyan fluorescent protein (CFP) and yellow fluorescent protein (YPet) were used as the donor and acceptor, respectively. Two AcrB fusion proteins were constructed, AcrB-CFP and AcrB-YPet. The proteins were co-expressed in *Escherichia coli* cells, and energy transfer efficiency were determined in live cells. To obtain reliable energy transfer data, a complete set of correction parameters need to be first determined to accommodate for factors such as background fluorescence and spectra overlap. This paper described the methodology and determination of the correction factors, which are useful data and reference points for researchers working on fluorescence measurement of membrane protein complexes in live bacteria cells. Further interpretation and discussion of these data can be found in “Comparison of in vitro and in vivo oligomeric states of a wild type and mutant trimeric inner membrane multidrug transporter” (Wang et al., in press).

**Specifications table**

**Table:** 

Subject area	*Biochemistry and biophysics*
More specific subject area	*Membrane protein fluorescence spectroscopy*
Type of data	*Graph, figure*
How data was acquired	*Zeiss LSM 880 with Airyscan laser scanning confocal microscope*
Data format	*Analyzed*
Experimental factors	*E. coli cells transformed with the indicated plasmid was cultured to the log phase and collected through centrifugation. The cell pellet was resuspended in phosphate buffer and dropped to a 1% agar disk supported on a microscopy cover slip. Cells were imaged directly without further treatment.*
Experimental features	*Protein of interest was tagged using a fluorescence protein domain, CFP or YPet. Energy transfer efficiency was measured using the spectrum-based fluorescence resonance energy transfer technique*
Data source location	*Lexington, KY, 40513, UA*
Data accessibility	*All data are presented in this article.*
Related research article	Z. Wang, W. Lu, P. Rajapaksha, T. Wilkop, Y. Cai, Y. Wei Comparison of in vitro and in vivo oligomeric states of a wild type and mutant trimeric inner membrane multidrug transporter, in press [1]

**Value of the data**

• The correction parameters determined and method described for the spectrum-based FRET can be used after adaptation by researcher conducting similar measurements.
• The correction method presented here eliminated the need of whole spectrum integration.
• The data can be used to reveal protein oligomeric state in live cell membranes.

## 1 Data

In this article, we present data and related method to obtain fluorescence energy transfer efficiency of a trimeric membrane protein AcrB in live bacteria cell using a spectrum-based FRET method. Such data are useful in the determination of protein-protein interactions, especially for membrane integrated proteins. Specifically, we first tagged AcrB with fluorescent proteins to construct AcrB-CFP and AcrB-YPet, and then we measured the FRET efficiency between CFP and YPet when the two fusion proteins were co-expressed in *Escherichia coli* cells. To determine the FRET efficiency, we first developed methodologies and determined a set of correction parameters.

**Fig. 1:**
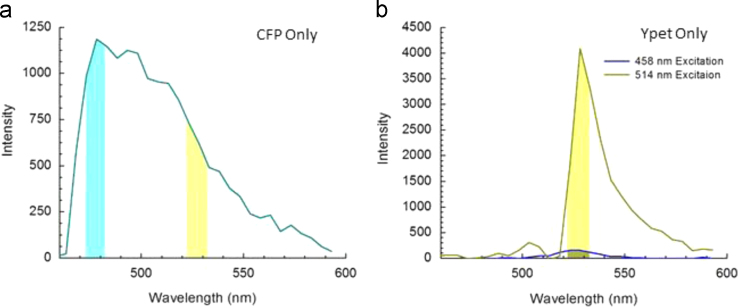
Representative emission spectra of AcrB-CFP (only acceptor labeled sample) (a) and AcrB-YPet (only donor labeled sample) (b). a) The blue and yellow bands indicate the spectral windows through the bandpass filter over which the fluorescence signals were recorded by the multichannel Airyscan detector. The intensities in these windows were used in the FRET measurements. The multichannel detector recorded the fluorescence signal between 473–483 nm (Channel 1, “C”) band, and the 523–533 nm (Channel 2 “F”) band using a 458 nm laser as the excitation source for imaging AraB-CFP. The ratio of the signals is used to calculate *CFP*_*BT*_. b) The YPet spectra were recorded under excitation with the 458 nm laser, blue line and a 514 nm laser, yellow line. The yellow region, of 523–533 nm (Channel 3, “Y”), indicates the transmission window for the Airyscan detector, when using either 514 or 458 nm laser line excitations. The respective areas under the curves are used to calculate *YPet*_*CE*_.

The fluorescent-labeled proteins are located in the *E. coli* membrane. In order to determine the emission spectra of the proteins, we performed an emission scan of the sample under excitation with the 458 nm laser and segmented the *E. coli* membrane in the microscope image for analysis. For robustness sake, and to maximize the signal to noise ratio in the measurement, the segmented regions of 20 cells were used to chart the representative spectral profile of the CFP and YPet, Fig. 1.

The spectra shown in Fig. 1 were used to determine correction factors. The maximum emission peaks for CFP and YPet are lying at 478 and 528 nm, respectively. CFP has significant emission near the YPet peak, and hence potential spectral bleed through has to be corrected for in the FRET efficiency calculation when the 458 nm laser is used. The CFP bleed through coefficient (*CFP*_*BT*_ ) is given by:

(1) $${\mathrm{CFP}}_{\mathrm{BT}}=\frac{\int_{523\;nm}^{533\;nm}I_{\mathrm{CFP}}^{458}\left(\lambda \right)d\lambda }{\int_{473\;nm}^{483\;nm}I_{\mathrm{CFP}}^{458}\left(\lambda \right)d\lambda }$$

where $I_{\mathrm{CFP}}^{458}\left(\lambda \right)$ is the CFP emission for excitation with the 458 nm laser (Fig. 1a). From the spectrum in Fig. 1a, *CFP_BT_* was determined as 0.556.

The YPet spectra in Fig. 1b are the fluorescence emission spectrum of the YPet only sample excited by 458 nm laser at 4% of its output power and by 514 nm laser at 1% of output power. The power setting of the lasers was the same for all the subsequent FRET characterizations. The YPet cross-excitation coefficient (*YPet*_*CE*_) is given by:

(2) $${\mathrm{YPet}}_{\mathrm{CE}}=\frac{\int_{523\;nm}^{533\;nm}I_{\mathrm{YPet}}^{458}\left(\lambda \right)d\lambda }{\int_{523\;nm}^{533\;nm}I_{\mathrm{YPet}}^{514}\left(\lambda \right)d\lambda }$$

where $I_{\mathrm{YFP}}^{458}\left(\lambda \right)$ is the YPet emission spectrum excited at 458 nm (the blue line in Fig. 1b), $I_{\mathrm{YFP}}^{514}\left(\lambda \right)$ is the YPet emission spectrum excited at 514 nm (the dark yellow line in Fig. 1b). From the two spectra in Fig. 1b, *YPet*_CE_ was determined as 0.046.

FRET determination *E. coli* cells expressing both AcrB-CFP and AcrB-YPet, or AcrB_P223G_-CFP and AcrB_P223G_-YPet were imaged in the channel mode. In channel 1 (C), the 458 nm laser at 4% power was used as the excitation source, with the fluorescence signal between 473 and 483 nm was recorded, in Channel 2 (F), the fluorescence signal between 523 and 533 nm was recorded and in channel 3 (Y), using the 514 nm laser at 1% power the fluorescence between 523 and 533 nm was recorded. For all channels the Airyscan detector was used with a gain setting of 800.

The fluorescence signal (*I*_*F*_) recorded in Channel 2 (F) contains three components:

(3) $$I_{F}=I_{\mathrm{FRET}}+I_{\mathrm{BT}}+I_{\mathrm{CE}}$$

The first component (*I*_*FRET*_) is the true FRET signal. The second component (*I*_*BT*_) is the CFP fluorescence signal that bleeds into the 523–528 nm band. *I*_*BT*_ is determined by the CFP fluorescence intensity *I*_*CFP*_ multiplied by the CFP bleed through coefficient (*CFP*_*BT*_). The third component is the YPet cross excitation (*I*_*CE*_). *I*_*CE*_ is calculated by multiplying the YPet fluorescence intensity at 514 nm (*I*_*YPet*_) with the YPet cross excitation coefficient (*YPet*_*CE*_).

$I_{F}$ can hence be re-write as:

(4) $$I_{F}=I_{\mathrm{FRET}}+I_{\mathrm{CFP}}\times {\mathrm{CFP}}_{\mathrm{BT}}+I_{\mathrm{YFP}}\times {\mathrm{YPet}}_{\mathrm{CE}}$$

This can be solved for the true FRET signal:

(5) $$I_{\mathrm{FRET}}=I_{F}-I_{\mathrm{CFP}}\times {\mathrm{CFP}}_{\mathrm{BT}}-I_{\mathrm{YFP}}\times {\mathrm{YPet}}_{\mathrm{CE}}$$

From the pure YPet emission spectrum *I_YPet_*(λ) plotted in Fig. 1b, the ratio between the overall emission intensity ($I_{\mathrm{FRET}}^{\mathrm{overall}}$) and the emission intensity between 523 and 533 nm (*I*_*FRET*_) was determined to be:

(6) $$R_{\mathrm{YPet}}=\frac{\int_{463\;nm}^{593\;nm}I_{\mathrm{YFP}}\left(\lambda \right)d\lambda }{\int_{523\;nm}^{533\;nm}I_{\mathrm{YFP}}\left(\lambda \right)d\lambda }=2.10$$

Similarly, from the pure CFP emission spectrum, the ratio of overall CFP emission intensity ($I_{\mathrm{CFP}}^{\mathrm{overall}}$) and the emission intensity recorded between 473 and 483 nm (*I*_*CFP*_) was determined to be:

(7) $$R_{\mathrm{CFP}}=\frac{\int_{463\;nm}^{593\;nm}I_{\mathrm{CFP}}\left(\lambda \right)d\lambda }{\int_{473\;nm}^{483\;nm}I_{\mathrm{CFP}}\left(\lambda \right)d\lambda }=4.63$$

The integration of spectral intensities over the range of 473–483 nm, or 523–533 nm, were used to calculate the emission intensity of the entire spectra for CFP and YPet, respectively. [2].

(8) $$E=\frac{I_{\lambda D}^{A}\cdot Q^{D}/Q^{A}}{I_{\lambda D}^{D}+I_{\lambda D}^{A}\cdot Q^{D}/Q^{A}}=\frac{I_{\mathrm{FRET}}R_{\mathrm{YPet}}\frac{Q_{\mathrm{CFP}}}{Q_{\mathrm{YPet}}}}{I_{\mathrm{CFP}}R_{\mathrm{CFP}}+I_{\mathrm{FRET}}R_{\mathrm{YPet}}\frac{Q_{\mathrm{CFP}}}{Q_{\mathrm{YPet}}}}$$

This yielded values for *Q*_*CFP*_ of 0.40 and *Q*_*YPet*_ of 0.77 [3], [4].

Populating the expression for *E* with the various correction factors thus yields.

(9) $$E=\frac{1.09(I_{F}-0.556I_{\mathrm{CFP}}-{0.046I}_{\mathrm{YPet}})}{{4.63I}_{\mathrm{CFP}}+1.09(I_{F}-{0.556I}_{\mathrm{CFP}}-{0.046I}_{\mathrm{YPet}})}$$

*With, I*_*CFP*_, *I*_*F*_, and *I*_*YPet*_ being the intensity values experimentally obtained for channel 1 (C), Channel 2 (F), and Channel 3 (Y), respectively.

## 2 Experimental design, materials, and methods

A Zeiss LSM 880 confocal microscope with Airyscan was used to image *E. coli* cells deposited on a gel pad, adopting the immobilization strategy outline in detail by Bottomley et al. [5]. The microscope can be operated in Airyscan mode, in which a 32 element detector is used to image the Airy disc, in this mode bandpass transmission filter are placed in the beam path, or it can be operated with photo multipliers that are picking up the fluorophore emissions over a user defined wavelength range or spectral scan. During our experiments both complementary modes were used. For standard imaging of the AcrB-CFP and YPet, the 458 nm and 514 nm laser line (at 1% power), respectively, were used for excitation, with the fluorescence emission being collected between 473–483 nm (CFP) and 523–533 nm (YPet), resulting in clear images of *E. coli* cells. AcrB is a membrane protein, the observed fluorescence signal was characteristically for a membrane protein, concentrated in an envelope -like profile in the *E. coli* cells.
